# Increased plant productivity and decreased microbial respiratory C loss by plant growth-promoting rhizobacteria under elevated CO_2_

**DOI:** 10.1038/srep09212

**Published:** 2015-03-18

**Authors:** Ming Nie, Colin Bell, Matthew D. Wallenstein, Elise Pendall

**Affiliations:** 1 Department of Botany and Program in Ecology, University of Wyoming, Laramie, WY 82071, USA; 2 Institute of Biological and Environmental Sciences, University of Aberdeen, Aberdeen, AB24 3UU, UK; 3 Natural Resource Ecology Laboratory, Colorado State University, Fort Collins, CO 80523, USA; 4 Hawkesbury Institute for the Environment, University of Western Sydney, Locked Bag 1797, Penrith, NSW 2751 Australia

## Abstract

Increased plant productivity and decreased microbial respiratory C loss can potentially mitigate increasing atmospheric CO_2_, but we currently lack effective means to achieve these goals. Soil microbes may play critical roles in mediating plant productivity and soil C/N dynamics under future climate scenarios of elevated CO_2_ (eCO_2_) through optimizing functioning of the root-soil interface. By using a labeling technique with ^13^C and ^15^N, we examined the effects of plant growth-promoting *Pseudomonas fluorescens* on C and N cycling in the rhizosphere of a common grass species under eCO_2_. These microbial inoculants were shown to increase plant productivity. Although strong competition for N between the plant and soil microbes was observed, the plant can increase its capacity to store more biomass C per unit of N under *P. fluorescens* addition. Unlike eCO_2_ effects, *P. fluorescens* inoculants did not change mass-specific microbial respiration and accelerate soil decomposition related to N cycling, suggesting these microbial inoculants mitigated positive feedbacks of soil microbial decomposition to eCO_2_. The potential to mitigate climate change by optimizing soil microbial functioning by plant growth-promoting *Pseudomonas fluorescens* is a prospect for ecosystem management.

Increased plant productivity and decreased microbial respiratory C loss could potentially mitigate increasing atmospheric CO_2_ concentrations, but we currently lack effective means to achieve these goals^1^,^2^,^3^. The accumulation of ecosystem C is controlled by the balance between plant productivity versus heterotrophic respiration through soil organic matter (SOM) decomposition^4^,^5^. Numerous studies have reported that elevated CO_2_ (eCO_2_) promotes plant growth and increases photosynthetic C input to soils^6^,^7^,^8^,^9^. These increased C inputs can stimulate microbial growth and N demand, which can limit soil N availability and plant N uptake under eCO_2_^10^. On the other hand, positive feedbacks of soil microbial communities under eCO_2_ may accelerate SOM decomposition and potentially result in soil net C losses^11^,^12^,^13^. It is clear that microbial dynamics play an important role in regulating net ecosystem C storage under future climates, but potentially counteracting responses make it difficult to determine their net effect.

Some beneficial microbial inoculants have been shown to increase plant growth by improving soil N availability in many managed ecosystems under ambient CO_2_^14^,^15^,^16^. However, stoichiometric homeostasis theory suggests that increased plant C fixation under eCO_2_ can accelerate soil decomposition related to N mineralization by increasing the allocation of resources towards the microbial production of enzymes that degrade N-rich substrates^17^,^18^,^19^. Recent studies suggest that eCO_2_ promotes SOM decomposition associated with microbial activity through increased rhizosphere priming effects (RPEs)^20^,^21^. Increased microbial utilization of C exudate under eCO_2_ can induce increased N mineralization and create positive nutrient feedbacks to mitigate soil N limitation^8^,^21^,^22^,^23^. However, in order to increase the efficiency by which microbes metabolize and transform plant detritus under eCO_2_, microbial inoculants should increase plant productivity, without accelerating SOM decomposition rates and microbial respiratory C loss. For example, applications of arbuscular mycorrhizal fungi (AMF) were generally shown to facilitate plant growth and stimulate soil carbon storage under ambient CO_2_^24^,^25^. However, AMF can speed up the turnover of recently fixed photosynthetic C associated with accelerated N cycling in spite of increased plant net primary productivity in forests exposed to eCO_2_, limiting soil C accumulation^20^,^21^.

Many isolated strains have been identified as plant growth-promoting rhizobacteria (PGPR), which could be used to stimulate plant growth under rising atmospheric CO_2_^26^,^27^. Numerous studies have shown that PGPR can effectively increase plant performance in nutrient-limited environments, through positively influencing root growth and morphology, and promoting other beneficial plant–microbe symbioses^28^,^29^. In various natural terrestrial ecosystems, application of PGPR inoculants has emerged as a technology to facilitate grassland management^30^, ecosystem restoration^29^ and reforestation^31^. In contrast to soil saprotrophic bacteria, PGPR appear to have high substrate use efficiencies^32^. Under future climate conditions, eCO_2_ may increase PGPR dominance as these plant-associated microbes enhance plant success under eCO_2_^26^,^33^. Nevertheless, our understanding of how plants interact with PGPR inoculants to regulate SOM decomposition and soil N availability in the rhizosphere is still limited, despite the potential of PGPB to alleviate environmental stresses imposed by eCO_2_^26^,^27^. The effective application of these PGPB in natural terrestrial ecosystems requires understanding the traits that may enhance ecological performance in the rhizosphere^3^,^16^.

Here, we used a dual-isotope labeling technique with ^13^C and ^15^N to evaluate the effect of *Pseudomonas fluorescens* inoculation on plant productivity and soil C/N cycling under eCO_2_. *P. fluorescens*, which is common in soils, improves plant growth through several different mechanisms, such as the suppression of plant diseases and enhancement of essential metal uptake^15^,^28^,^31^. The objectives of the current study were to test whether using *P. fluorescens* as a soil microbial inoculum (1) improves plant productivity and photosynthetic C input to soils, (2) promotes plant N use, and (3) mitigates C lost through microbial respiration.

## Results

**Figure 1:**
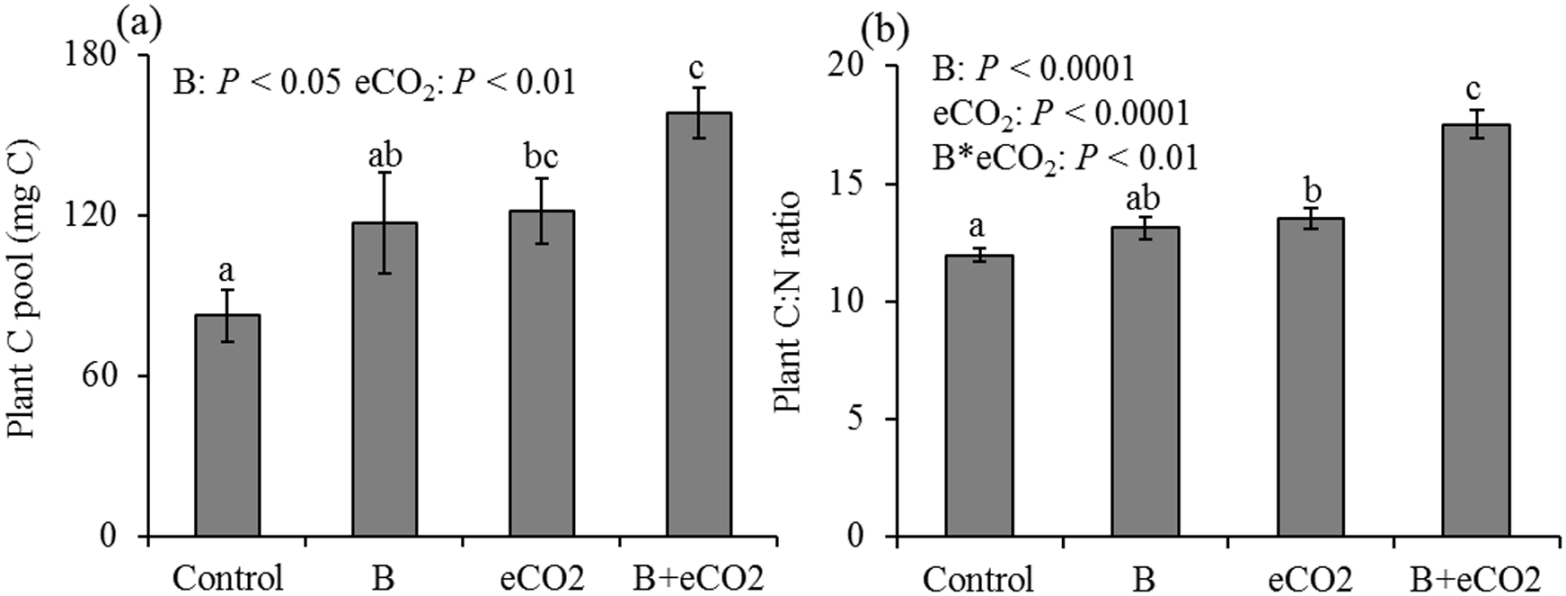
Plant C pool size per pot (a) and biomass C:N ratio (b). Control: ambient CO_2_ and without bacteria addition; B: ambient CO_2_ and with bacteria addition; eCO_2_: elevated CO_2_ and without bacteria addition; B + eCO_2_: elevated CO_2_ and with bacteria addition. Error bars show standard error of the mean (n = 6). The same letters denote non-significant differences between treatments (*P* > 0.05).

**Figure 2:**
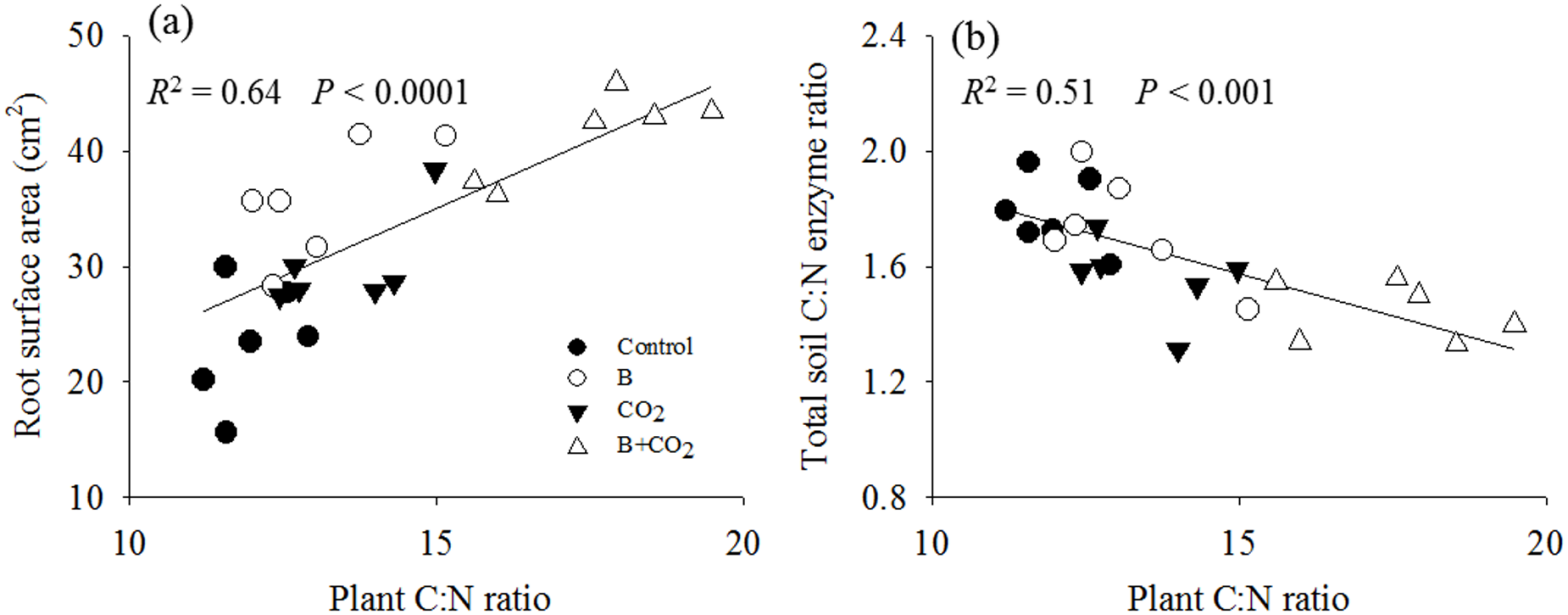
Linear relationships of plant C:N ratio with root surface area (a) and total C:N enzyme ratio (b) across all treatments. Control: ambient CO_2_ and without bacteria addition; B: ambient CO_2_ and with bacteria addition; eCO_2_: elevated CO_2_ and without bacteria addition; B + eCO_2_: elevated CO_2_ and with bacteria addition.

Bacteria and eCO_2_ had positive, cumulative effects on plant productivity in terms of total plant biomass C (Figure 1a). The highest plant productivity occurred in the combined bacteria and eCO_2_ treatment (Figure 1a). In addition, bacteria and eCO_2_ increased the plant C:N ratio as individual treatments, but demonstrated a synergistic effect when combined (Figure 1b), suggesting that plants can assimilate more C per unit of N in tissue under eCO_2_ with these *P. fluorescens* inoculants. Increased plant root surface area associated with bacterial inoculants and eCO_2_ (Table S1) demonstrated a strong positive relationship with plant tissue C:N (Figure 2a). Combined bacterial inoculant and eCO_2_ treatments demonstrated significantly lower total soil enzyme C:N acquisition activity ratios (Table S1); and decreases in enzyme C:N were strongly correlated with higher plant tissue C:N (Figure 2b), suggesting a strong competition for N between plant and soil microbes.

**Figure 3:**
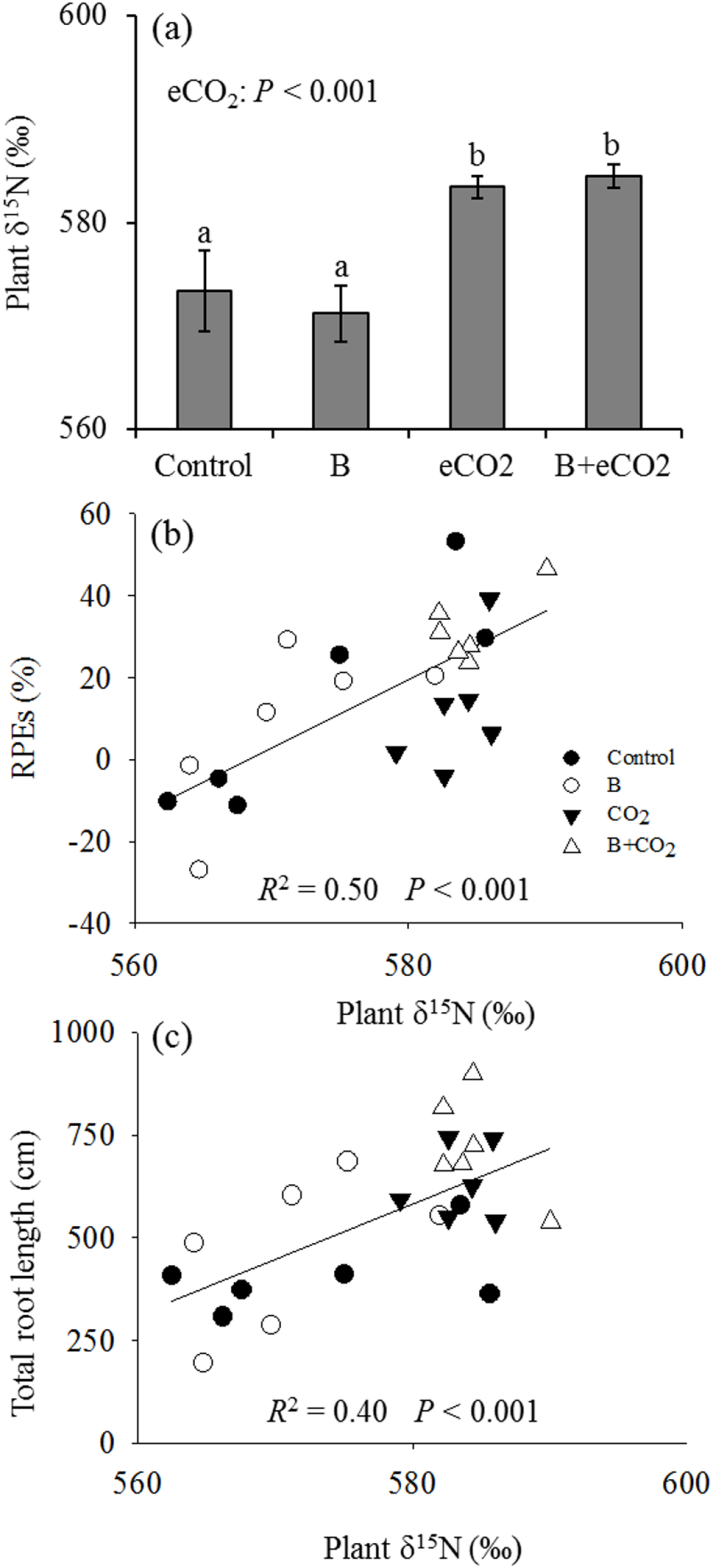
δ^15^N value in plant biomass (a), and linear relationships of plant δ^15^N value with rhizosphere priming effects (RPEs) (b) and total root length (c) across all treatments. Control: ambient CO_2_ and without bacteria addition; B: ambient CO_2_ and with bacteria addition; eCO_2_: elevated CO_2_ and without bacteria addition; B + eCO_2_: elevated CO_2_ and with bacteria addition. Error bars show standard error of the mean (n = 6). The same letters denote non-significant differences between treatments (*P* > 0.05).

We took advantage of the distinct δ^15^N values to estimate the relative sources of plant N (N from the original inorganic pool vs. N mineralized from SOM). eCO_2_ significantly increased the δ^15^N value in plant biomass (Figure 3a). The higher δ^15^N in soil organic N compared to soil inorganic N (see δ^15^N values of two pools in Methods) indicates that eCO_2_ increased plant uptake of mineralized N from SOM relative to soil inorganic N. However, *P. fluorescens* inoculants did not change plant δ^15^N values at each CO_2_ level (Fig. 3a), suggesting these inoculants did not accelerate soil decomposition related to N mineralization.

Although high variability in rhizosphere priming effects was observed among the treatments, priming of SOM decomposition was positively related with plant δ^15^N (Figure 3b), suggesting that rhizosphere exudation (priming) was induced by plants in order to facilitate N mineralization from SOM. Likewise, total root length (which determines root system expansion) was positively related with plant δ^15^N (Figure 3c).

**Figure 4:**
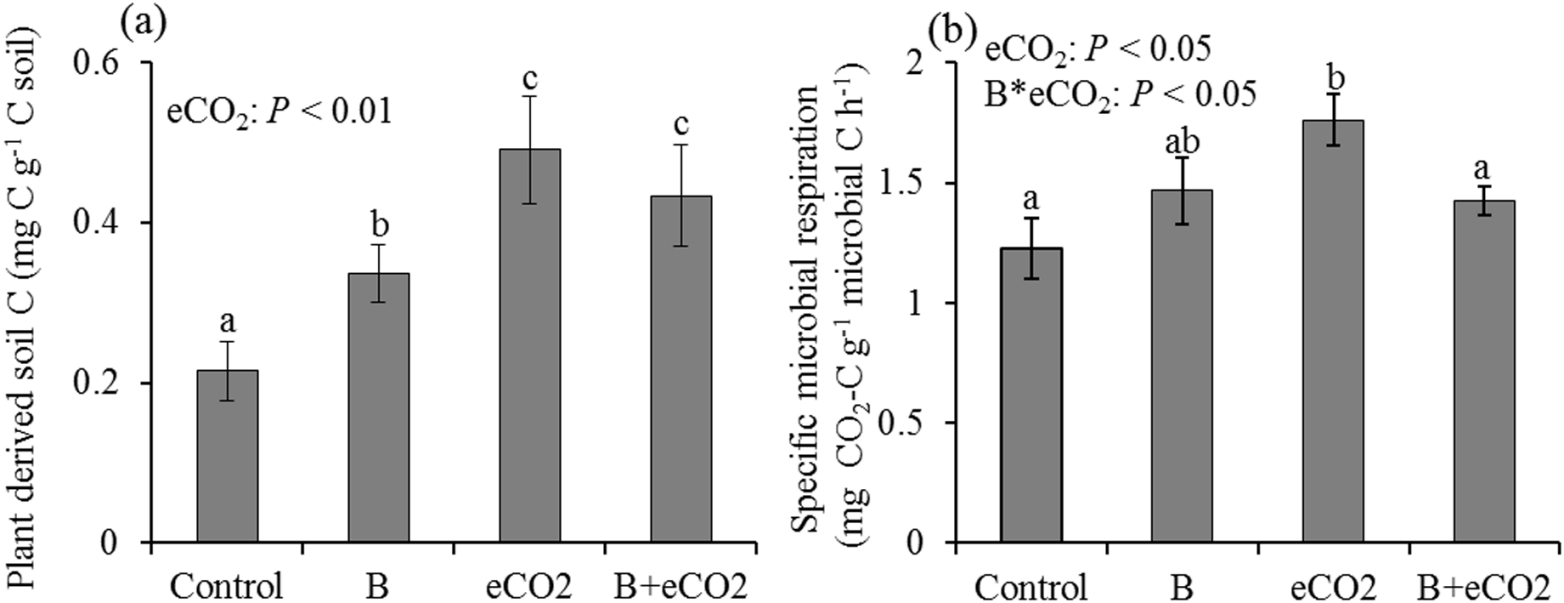
Plant-derived C inputs to soil (a) and specific microbial respiration (b). Control: ambient CO_2_ and without bacteria addition; B: ambient CO_2_ and with bacteria addition; eCO_2_: elevated CO_2_ and without bacteria addition; B + eCO_2_: elevated CO_2_ and with bacteria addition. Error bars show standard error of the mean (n = 6). The same letters denote non-significant differences between treatments (*P* > 0.05).

Compared to the control, all treatments induced higher soil C inputs from the plant-derived C (Figure 4a). However, significantly higher mass-specific microbial respiration (lower C use efficiency) was observed only under eCO_2_ (Figure 4b).

## Discussion

The capability of plants and soil microbes to successfully sequester atmospheric C in terrestrial ecosystems largely depends on plant productivity along with microbial decomposition and mineralization feedbacks within the rhizosphere^8^,^10^,^11^,^12^. Advancing current efforts to mitigate effects of climate change could minimize harmful effects of elevated CO_2_^1^,^26^. Here, for the first time, we show that addition of a microbial inoculant has the potential to promote plant productivity while mitigating positive feedbacks of microbial decomposition to increased plant C inputs that typically accompany eCO_2_. Our results also demonstrated that *P. fluorescens* inoculation led to increased plant tissue C:N under eCO_2_, resulting in an increased capacity to store C per unit of N in plant tissue. Therefore, this soil microbial inoculant may be a useful tool to mitigate climate change.

The results from this study are consistent with numerous field and growth chamber experiments showing that *P. fluorescens* inoculants can increase plant production under ambient CO_2_^14^,^15^,^31^, and show that these largely stimulating effects could be additive when combined with eCO_2_. Although it was impossible to exclude potential fertilization effects on plant available N through bacterial cell addition, the increases in total soil N pool by bacterial cell addition (0.016%) is likely negligible in comparison with the increase in plant growth by an average of 42%. In addition, chemical adjustments to litter C:N may contribute to reduced quality and decomposability of plant litter under eCO_2_^9^,^34^ which may reduce soil decomposition rates. In comparison to our previous study, overall plant C:N ratios during the rapid vegetative growth stage were lower than during the reproductive stage^35^.

Plant roots play an essential role in regulating acquisition of soil nutrients. Nevertheless, little is known about the relationship of root functional traits with plant N use strategies. Root surface area is generally correlated to plant nutrient uptake rates^36^. *P. fluorescens* inoculants significantly increased root surface area under eCO_2_ (Table S1), suggesting that *P. fluorescens* inoculants could enhance the potential for plant roots to acquire N under N-limited eCO_2_ condition. Moreover, our results demonstrated that root surface area positively correlated with plant C:N ratio across experimental treatments (Figure 2a), further indicating that the plants' ability to acquire N from the soil could be influenced by soil N availability.

Coupled C and N processes in the rhizosphere play a critical role in maintaining the sustainability of ecosystems^34^,^37^. Recent studies suggest that plant productivity slows when plant N demand decouples from soil N cycling under climate change or other ecological disturbances^38^,^39^. In our previous study conducted in the same ecosystem as this present work, we found that eCO_2_ increased microbial biomass N immobilization and decreased soil N availability^19^. Likewise, this study revealed that eCO_2_ significantly decreased soil enzyme C:N stoichiometry (Table S1), indicating greater microbial demand for soil N under eCO_2_^40^. N limitation could ultimately dampen ecosystem C sequestration in terms of the eCO_2_ fertilization effect on plant productivity^10^,^34^. However, *P. fluorescens* inoculant did not directly affect soil enzyme C:N stoichiometry (Table S1). Moreover, the negative relationship between plant biomass C:N and enzyme C:N (Figure 2b) suggests that plants can continue to grow through increase in their capacity to store C per unit of N in response to changes in soil N availability, which is mediated by soil microbial activities.

It is well known that plants can alter their N uptake rates to cope with plant physiological and environmental changes^41^,^42^. By using the ^15^N isotopic method, we observed that eCO_2_ increased the importance of N mineralized from SOM, indicating that the positive effects of eCO_2_ on soil N enzyme activities increased soil N availability (Figure 3a and Table S1). However, *P. fluorescens* inoculants had no detectable effects on δ^15^N values of plant biomass (Figure 3a) or on soil N enzyme activities (Table S1). This suggests that *P. fluorescens* does not facilitate N mineralization under eCO_2_ conditions. We note that N in *P. fluorescens* cells is mostly in organic form with the δ^15^N value of 4.2‰ (the δ^15^N value of organic N in SOM is 587.5‰). If plants took up mineralized N from dead *P. fluorescens* cells, the δ^15^N value of plant biomass should be lower than plant uptake. However, even if all bacterial N was absorbed by plants, the N in *P. fluorescens* cells would only contribute from 0.8% to 2.2% of the total plant N pool. In addition, the δ^15^N value of organic N corrected by bacterial cells (587.4‰) was still much higher than inorganic N (445.7‰).

eCO_2_-induced rhizosphere priming effects and subsequent microbial N mineralization could influence the magnitude of plant growth^8^,^21^. Previous studies have identified several plant and microbial traits related to RPE^8^,^21^,^43^, but the direct evidence of priming-related effects on plant N availability has not been well documented. Our results, for the first time, clearly demonstrated that priming of SOM decomposition was positively related to plant N availability (Figure 3b), suggesting that priming made soil N more available to the plant for uptake. Increased root length was also observed in this study as an important root functional trait related to plant N uptake adaptations associated with microbial plant growth-promoting properties under eCO_2_ conditions. These results add to a growing body of evidence that plants could increase N availability through rhizosphere priming and development of root systems to alleviate nitrogen limitation under eCO_2_^8^,^9^,^21^,^43^.

*P. fluorescens* inoculants and eCO_2_ were expected to increase plant C inputs to soil (Figure 4a). However, a synergistic effect of bacteria and eCO_2_ on plant-derived C was not observed (Figure 4a). This may be due to the use of planting pots which may have constrained root growth in this experiment. Results from a meta-analysis suggest that CO_2_-induced increases in belowground biomass are stronger in plants grown in open fields relative to closed pots^9^. In spite of higher rates of new C inputs, *P. fluorescens* inoculants and eCO_2_ demonstrated contrasting effects on heterotrophic respiration due to microbial activities. eCO_2_ increased mass-specific microbial respiration (Figure 4b)^35^, consistent with previous observations of climate-induced positive feedbacks^12^,^40^. *P. fluorescens* inoculants did not change mass-specific microbial respiration under ambient CO_2_ but mitigated positive microbial feedbacks under eCO_2_ conditions (Figure 4b). Thus, these findings suggest that *P. fluorescens* inoculants may potentially decrease soil C losses via heterotrophic respiration.

Our results indicate that *P. fluorescens* inoculants may optimize soil microbial functioning and potentially be implemented as a strategy for increasing plant productivity while mitigating positive feedbacks of microbial decomposition to eCO_2_. If the benefits of *P. fluorescens* inoculants can be scaled from the growth chamber and applied in natural ecosystems in a high-CO_2_ world, the potential for terrestrial C sequestration may increase to mitigate rising atmospheric CO_2_. Further assessment is needed to extend these findings to field experiments and to formulate economical methods of inoculation for field deployment. Additional experiments should be performed to assess rhizosphere colonization by *P. fluorescens* inoculants across a range of plant species.

## Methods

### Experimental setup

The C4, perennial grass *Bouteloua gracilis* was selected in this experiment because it is a widespread grass in North America and accounts for most ecosystem net primary productivity in the shortgrass prairie of the central and southern Great Plains^44^. We collected soils for this experiment from the USDA-ARS Central Plains Experimental Range, Colorado, USA. To trace sources of plant N uptake in our experiment, we took advantage of ^15^N-enriched soil from a prior experiment^43^. About 15 years after 0.5 g m^−2^ ^15^N was added (and 9 years after the completion of the original experiment), soil was collected from the top 15-cm and sieved (mesh size 2 mm) to remove roots and homogenize the soil. The soil is a Remmit fine sandy loam (Ustollic camborthids) with 0.8% organic C in the top 15 cm. To reduce soil nutrient availability, we leached the soil with DI water in large buckets with small waterspouts. After that, the soil was air-dried and passed through a 2-mm sieve to further remove plant residues, soil fauna and other coarse materials, and then homogenized to attain a composite sample. 600 g soil (dry weight) was packed into each plastic pot at a similar bulk density to field conditions. The pots were capped at the bottom and no leaching occurred during the experiment. Before our experiment, the initial soil inorganic N (NH_4_^+^ + NO_3_^−^) and organic N (total soil N minus soil inorganic N) content were 0.14 ± 0.002 and 0.41 ± 0.03 mg g^−1^, respectively (n = 8; t-statistic < 0.0001). The δ^15^N values of inorganic and organic N were 445.7 ± 12.5 and 587.5 ± 10.8‰, respectively (n = 8; t-statistic < 0.0001). Inorganic δ^15^N was determined using the diffusion method^45^. Organic N was estimated as the difference between total and inorganic N, and organic δ^15^N was determined by mass balance. The δ^15^C value of soil organic matter was −20.4‰ and no inorganic C was present.

We performed our experiment in climate-controlled growth chambers (Percival PGC-9/2, Percival Scientific, Perry, IN, USA). The chamber systems we used were shown to have high reliability and stability during comparative studies of plant genetics and eco-physiology^46^,^47^. To simulate field conditions during the growing season, the growth chambers were set to a 14 h daytime period with light intensity of 700 μmol m^−2^ s^−1^. The daytime and night-time temperatures were 25°C and 18°C, respectively. We used a Li-250 light meter (LI-COR, Lincoln, NE, USA) and Telaire 7001 meters (Telaire, Goleta, CA, USA) to ascertain the reliability of light intensities and temperatures of the chambers every day. To achieve continuous ^13^C-labeling of plant tissues, the chambers were modified to receive an influx of ^13^C-depleted CO_2_ (δ^13^C = −33.1‰) combined with an external air input which had been scrubbed by a 70-L gas tight soda lime column. The CO_2_ concentrations inside the chambers were calibrated by infrared CO_2_ sensors (GMM220, Vaisala, Helsinki, Finland) and continuously monitored by Telaire 7001 meters (ambient CO_2_ concentration: 371.9 ± 2.1 ppm (mean ± se)); elevated CO_2_ concentration: 702.9 ± 8.7 ppm). The δ^13^C values of CO_2_ inside chambers were continuously monitored by a Picarro G2101i ^13^CO_2_ analyzer (Picarro, Sunnyvale, CA, USA). Throughout the experiment, the δ^13^C values of CO_2_ were stable (ambient CO_2_: −25.0 ± 0.2‰; elevated CO_2_: −24.9 ± 0.2‰), and there was no significant daily difference in the δ^13^C values of experimental chambers.

We used 24 planted pots with six replicates for each treatment: ambient CO_2_ without bacteria addition (Control), ambient CO_2_ and with bacteria addition (B), elevated CO_2_ and without bacteria addition (eCO_2_); elevated CO_2_ and with bacteria addition (B + eCO_2_). Correspondingly, another 24 unplanted pots were set up with six replicates for each treatment. Three seedlings were transferred to each planted pot after the emergence of the first euphylla on moist filter paper in glass Petri dishes. During the first week of the experiment, all pots (including unplanted plots) were rewetted to 25% gravimetric soil moisture content to enhance seedling growth. After that, the gravimetric water content in each pot was maintained at 15% (approximately 50% water holding capacity) using DI water, with no fertilizer additions. On day 10, we inoculated 1.5 × 10^9^ cells of *P. fluorescens* isolated from soil to 24 randomly assigned pots (12 planted and 12 unplanted), which we refer to as bacterial treatments. The strains were grown in liquid LB medium (Sigma, USA) at room temperature on a rotary shaker (200 rpm). After 48 h the cells were harvested by centrifugation for 10 min at 7000 rpm at 4°C and re-suspended in sterile DI water. Following two additional washes, they were re-suspended in sterile DI water. To obtain 1.5 × 10^9^ cells, the cell density was adjusted based upon optical density measured at 600 nm^48^. Bacterial cell addition only increased the total soil C and N pool sizes by 0.006% and 0.016%, respectively. The δ^13^C and δ^15^N values of bacterial cells were −24.9‰ and 4.2‰, respectively. After bacterial cell addition, therefore, the δ^13^C value of soil organic matter and δ^15^N value of soil organic N was −20.4‰ and 587.4‰, respectively.

### Measurements

We harvested 30 days after planting, because plants have high rates of interactions with soil processes during the rapid vegetative growth stage^35^,^49^. We placed each pot (planted and unplanted pots) in an opaque, capped PVC chamber (45-cm height, 20-cm diameter)^50^. Briefly, we sealed the bottom of chamber by placing it on a plastic dish containing water to impede gas loss, and removed CO_2_ inside each chamber by circulating air through a gas tight in-line soda-lime scrubber for 30 min. Then we immediately collected an initial 30 ml air sample with a plastic syringe to ensure CO_2_ scrubbing was complete. After 3 h we collected a final CO_2_ sample. Half of the final gas sample was analyzed for δ^13^C by a Thermo Finnigan Delta Plus XP isotope ratio mass spectrometer (Thermo Finnigan, Bremen, Germany) and the other was analyzed for CO_2_ concentration by a Li-Cor 820 (LICOR Inc. Lincoln, NE) calibrated with 4 standard gases.

After CO_2_ trapping, we immediately separated plants into shoots and roots, and homogenized soils. Traits of fresh roots (root length and surface area) were analyzed by a WinRHIZO system (Regent Instruments, Montreal, QC, Canada). Then each plant tissue and part of each soil sample were dried, weighed, ground, and analyzed for C/N concentrations and ^13^C/^15^N by a Thermo Finnigan Delta Plus XP isotope ratio mass spectrometer.

The continuous ^13^C-labeling of plant tissues allowed us to separate total soil respiration (*C_total_*) into SOM-C (SOC) decomposition (*C_SOC_*) and root respiration using the following model^51^:
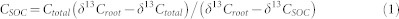
where *δ*^*13*^*C_root_*, *δ*^*13*^*C_total_* and *δ*^*13*^*C_SOC_* are the *δ*^*13*^*C* values of the root respiration, the total soil respiration in the planted treatments and the mean value of soil respiration in the corresponding unplanted treatments, respectively. For each CO_2_ treatment, the *δ*^*13*^*C* value of the root respiration was determined by growing plants in a SOM-free sand.

We calculated rhizosphere priming effects (RPEs) using following equation:
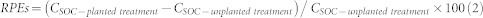


We calculated the amount of new soil C (*C_new_*) from plant-derived C through rhizodeposition during the experiment using the following model^52^:
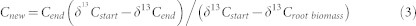
where *C_end_* is total amount of SOC at the end of the experiment, *δ*^*13*^*C_start_* (corrected by the C in the inoculants if needed) and *δ*^*13*^*C_end_* each are the *δ*^*13*^*C* values of SOC at the start and end of the experiment, and *δ*^*13*^*C_root_ _biomass_* is the *δ*^*13*^*C* values of root biomass.

A subsample of fresh soil from each pot was used to assess microbial community attributes. To normalize activity to the size of the microbial community, specific microbial respiration was calculated as the ratio of SOC decomposition rate (*C_SOC_*) to microbial biomass C (MBC)^35^. MBC was determined by the fumigation–extraction method and the factor for MBC calculation was 0.45^53^. To stoichiometrically link plant nutrient availability and microbial-mediated SOM decomposition, we measured soil enzyme activities involved in the cycling of C (β-Glucosidase and β-D-Cellubiosidase) and N (N-acetyl-β-Glucosaminidase and Leucine amino peptidase) cycling. The enzyme activities were measured using a 4-methylumbelliferyl (MUB) substrate yielding the highly fluorescent cleavage products MUB upon hydrolysis^40^.

### Statistical analyses

To determine the effects of eCO_2_ and bacteria on plant productivity and soil C/N cycling, we used a two-way ANOVA with eCO_2_ and bacteria as fixed effects by SPSS 13.0. Post hoc means were determined using least squares means separation by SPSS. The significance level (*P* value) of post-hoc LSD (least significant difference) test was set to 0.05. Data not meeting assumptions of normality and homogeneity of variance were log-transformed before statistical testing. Simple regression was performed to evaluate relationships underlying rhizosphere processes by Sigma Plot 10.0. Significant effects are reported at *P* < 0.05 unless otherwise stated.

## Supplementary Material

Supplementary Information Table S1
