# Supplementary material for: Increased plant productivity and decreased microbial respiratory C loss by plant growth-promoting rhizobacteria under elevated CO2
Source: Sci Rep. 2015 Mar 18;5:9212. doi: 10.1038/srep09212 (PMC4363858; doi:10.1038/srep09212)
Supplement: Supplementary Information — Table S1 [file srep09212-s1.docx]

**Increased plant productivity and decreased microbial respiratory C loss by**

**plant growth-promoting rhizobacteria under elevated CO_2_**

Ming Nie^1,2*^, Colin Bell^3^, Matthew D. Wallenstein^3^ & Elise Pendall^1,4^

^1^Department of Botany and Program in Ecology, University of Wyoming, Laramie, WY 82071, USA, ^2^Institute of Biological and Environmental Sciences, University of Aberdeen, Aberdeen, AB24 3UU, UK, ^3^Natural Resource Ecology Laboratory, Colorado State University, Fort Collins, CO 80523, USA, ^4^Hawkesbury Institute for the Environment, University of Western Sydney, Locked Bag 1797, Penrith, NSW 2751 Australia.

* Corresponding author

**Table S1 Plant root traits and soil enzyme activities**

|  | Root surface area (cm^-2^) | Total root length (cm) | Soil C enzyme activities (nmol h^−1^ g^−1^ dry soil) | Soil N enzyme activities (nmol h^−1^ g^−1^ dry soil) | Total soil C:N enzyme ratio |
| --- | --- | --- | --- | --- | --- |
| Control | 23.5 (2.1) | 406.9 (37.8) | 173.1 (5.3) | 97.1 (2.0) | 1.8 (0.1) |
| B | 35.7 (2.1) | 468.1 (77.4) | 167.4 (10.1) | 96.2 (2.0) | 1.7 (0.1) |
| eCO2 | 30.1 (1.7) | 631.4 (77.0) | 155.3 (4.7) | 100.0 (2.9) | 1.6 (0.1) |
| B+eCO2 | 41.4 (1.5) | 722.3 (51.0) | 155.9 (7.4) | 108.2 (5.5) | 1.4 (0.04) |
| *ANOVA p-values* |  |  |  |  |  |
| B | 0.0000 | 0.1694 | 0.7275 | 0.2975 | 0.1876 |
| eCO2 | 0.0039 | 0.0002 | 0.0552 | 0.0434 | 0.0003 |
| B+eCO2 | 0.8216 | 0.7843 | 0.6686 | 0.2002 | 0.5951 |

Values are means with standard errors in parentheses (n = 6). Control: ambient CO_2_ and without bacteria addition; B: ambient CO_2_ and with bacteria addition; eCO_2_: elevated CO_2_ and without bacteria addition; B+eCO_2_: elevated CO_2_ and with bacteria addition
